# Sleep disorders as both risk factors for, and a consequence of, stroke: A narrative review

**DOI:** 10.1177/17474930231212349

**Published:** 2023-11-20

**Authors:** Lukas Mayer-Suess, Abubaker Ibrahim, Kurt Moelgg, Matteo Cesari, Michael Knoflach, Birgit Högl, Ambra Stefani, Stefan Kiechl, Anna Heidbreder

**Affiliations:** 1 Department of Neurology, Medical University of Innsbruck, Innsbruck, Austria; 2 VASCage—Research Centre on Clinical Stroke Research, Innsbruck, Austria; 3 Neurological Clinical Research Institute, Massachusetts General Hospital, Boston, MA, USA; 4 Department of Neurology, Johannes Kepler University Linz, Linz, Austria

**Keywords:** Stroke, sleep, insomnia, narcolepsy, RBD, RLS, PLMS, prevention

## Abstract

**Background and purpose:**

Sleep disorders are increasingly implicated as risk factors for stroke, as well as a determinant of stroke outcome. They can also occur secondary to the stroke itself. In this review, we describe the variety of different sleep disorders associated with stroke and analyze their effect on stroke risk and outcome.

**Methods:**

A search term-based literature review (“sleep,” “insomnia,” “narcolepsy,” “restless legs syndrome,” “periodic limb movements during sleep,” “excessive daytime sleepiness” AND “stroke” OR “cerebrovascular” in PubMed; “stroke” and “sleep” in ClinicalTrials.gov) was performed. English articles from 1990 to March 2023 were considered.

**Results:**

Increasing evidence suggests that sleep disorders are risk factors for stroke. In addition, sleep disturbance has been reported in half of all stroke sufferers; specifically, an increase is not only sleep-related breathing disorders but also periodic limb movements during sleep, narcolepsy, rapid eye movement (REM) sleep behavior disorder, insomnia, sleep duration, and circadian rhythm sleep–wake disorders. Poststroke sleep disturbance has been associated with worse outcome.

**Conclusion:**

Sleep disorders are risk factors for stroke and associated with worse stroke outcome. They are also a common consequence of stroke. Recent guidelines suggest screening for sleep disorders after stroke. It is possible that treatment of sleep disorders could both reduce stroke risk and improve stroke outcome, although further data from clinical trials are required.

## Introduction

Cardiovascular disease (CVD) remains the driving factor in all-cause mortality globally and represents the leading cause of long-term morbidity in the Western world.^1^ Sleep disorders like sleep-related breathing disorders (SRBD) and insomnia are highly prevalent in modern society and have been increasingly recognized as significant contributors to the development and progression of CVD, including stroke.^2^ In this context, SRBD affects 20% of the general population, 50–60% of stroke survivors and increase the risk of incident, recurrent stroke as well as worse functional outcome.^3,4^ Recent reviews and expert statements have compiled the current evidence and discussed future perspectives of SRBD in cerebrovascular disease.^3,4^ However, there are several other frequent nonrespiratory sleep disorders which affect stroke risk and can manifest after stroke. The importance of sleep health in the primary stroke prevention has been postulated years ago. Current European guidelines recommend screening and treatment of sleep apnea in the acute phase of stroke; however, stroke care services have not yet implemented these recommendations.^3^ Therefore, the goal of the current article is to shed light on the current evidence of an association between sleep disorders and stroke.

## SRBD

Obstructive sleep apnea (OSA) is one of the most neglected risk factors for stroke with a prevalence of about 20% and doubled risk of stroke, especially in young/middle-aged adults, if untreated. OSA is also linked to wake-up stroke. Possible pathomechanisms include nondipping blood pressure during sleep, hypoxemia, impaired cerebral hemodynamic and a hypercoagulable state with elevated hematocrit, viscosity, and altered platelet function. Furthermore, OSA is one of the most common reasons for therapy refractive hypertension and has been linked to atrial fibrillation and cerebral microangiopathy.^4,5^ In addition, it is commonly associated with increased levels of depression and anxiety.^6^ On the contrary, as a meta-analysis of five observational studies presented, continuous positive airway pressure (CPAP) therapy is associated with stroke-risk reduction (27%) in primary prevention. Still, other studies (one randomized controlled trial (RCT), 2 health administrative data based) showed equivocal data. In secondary stroke prevention, a meta-analysis of RCTs could not demonstrate a reduction in stroke risk; however, a limitation was the poor CPAP compliance (3.3 h/night). In a post hoc analysis of those using CPAP for more than 4 h/night, treatment was associated with risk reduction. An excellent detailed review on sleep apnea and ischemic stroke by the group of C. Bassetti was recently published.^4^

## Nonrespiratory sleep disorders as a risk factor for stroke

### Restless legs syndrome and periodic limb movements during sleep

Restless legs syndrome (RLS) is frequent but still under-recognized, as emphasized by the population-based Bruneck study (10.8% prevalence of undiagnosed and untreated RLS).^7^ It is characterized by unpleasant sensations and an urge to move (mainly the legs) primarily at rest or during inactivity. These sensations are relieved by movement. As the symptoms typically occur in the evening or at night, RLS can cause sleep disturbances, which may be severe.^8–10^ Most patients with RLS present accompanying periodic limb movements in sleep (PLMS), characterized by limb movement of 15/h in adults according to the American Academy of Sleep Medicine scoring manual.^8^ PLMS are, however, not exclusively present in RLS patients, as they can occur in other sleep disorders and medical conditions or in healthy subjects.^11,12^ RLS has been linked to an increased risk of diabetes, hypertension, and a higher probability of being obese^13,14^ while PLMS have been associated with hypertension.^15,16^ The Wisconsin Sleep Cohort and the Sleep Heart Health Study reported a significant correlation between RLS and CVD including stroke.^17^ In a recent systematic review with meta-analysis, RLS patients have a higher risk of stroke and mortality but after adjustment for confounders, only the mortality association remained significant. In large meta-analyses on PLMS patients (five studies, 9823 PMLS patients/9.416 controls), stroke risk was increased by about 25% within 8 years after diagnosis.^3,18^ Concerning mechanisms connecting RLS/PLMS and CVD, inflammation,^19^ sympathetic activation,^20,21^ metabolic dysregulation,^13^ and hypothalamic–pituitary–adrenal system activation^14^ seem to play a role, but a definite pathway remains elusive.^22^

### Central disorders of hypersomnolence (Narcolepsy)

The key subjective complaint of central disorders of hypersomnolence, one of which is narcolepsy, is excessive daytime sleepiness. Patients report daily episodes of irrepressible urges to sleep, and lapses into sleep, which can be objectified in polysomnography and multiple sleep latency testing (MSLT) showing an increased readiness to fall asleep during the day (<8 min).^8^ Globally, the estimated prevalence of narcolepsy is 25–50 in 100,000. A distinction is made into (1) narcolepsy type I, with characteristic hypocretin-1 deficiency, increased readiness to fall asleep with sleep-onset rapid eye movement periods (SOREMPs) during polysomnography (PSG)/MSLT and clear cataplexy or (2) narcolepsy type II, with at least two SOREMPs.^8,23^

Studies on stroke risk in patients with narcolepsy remain scarce. One retrospective analysis (Burden of Narcolepsy Disease (BOND) study) investigated 9312 individuals with type 1 and 2 narcolepsy and revealed an odds ratio (OR) of 2.5 (2.3–2.7) to suffer stroke compared with matched controls.^24^ Mechanistically, hypocretin is believed to be involved in autonomic function and control (i.e. hypertension) relating to its plausible causative connection to CVD risk.^24,25^ Supporting this hypothesis, McAlpine et al.^26^ found that in apolipoprotein E knockout mice subjected to sleep deprivation and fragmentation, the hypothalamus produced less hypocretin which increased the likelihood of atherosclerotic lesions. They argued that hypocretin modulates the release of colony-stimulating factor 1 in the bone marrow, which regulates monocyte production, inflammation, and atherosclerosis.^26^ Still, further clinical studies are needed to confirm and elucidate mechanisms underlying this link.^27^

### Rapid eye movement sleep behavior disorder

Rapid eye movement (REM) sleep behavior disorder (RBD) is characterized by dream enactment behaviors (movement or vocalization) occurring during REM sleep and by the loss of the physiological REM sleep atonia, demonstrated by polysomnography.^8,28^ It is recognized as a prodromal synuclein-related neurodegenerative disease, as most (>90%) patients with isolated RBD (iRBD) phenoconvert to Parkinson’s disease, dementia with Lewy bodies or, less frequently, multiple system atrophy 15 years or more after onset.^8,29–31^ One large community-based questionnaire study (n > 12,000) of probable RBD patients reported an increased risk for ischemic (hazard ratio (HR) = 1.93 (1.07–3.46) and hemorrhagic stroke (HR = 6.61 (2.27–19.27)^32^ As autonomic nervous system function is frequently impaired in patients with iRBD (e.g. nondipping profile in 24 h blood pressure), the connection to higher risk of CVD is feasible.^33^ Still, current evidence is low making future prospective studies in well-characterized cohorts with definite iRBD necessary to establish the potential relationship.

### Insomnia, sleep duration, and circadian rhythm sleep–wake disorders

Insomnia, defined by difficulty in sleep initiation/maintenance and early awakening with daytime consequences at least three times per week over a span of at least 3 months, is one of the most frequent sleep disorders with approximately one-third of the general population reporting attributable features.^8,34^ Insomnia is linked to metabolic syndrome,^35^ hypertension^36^ and depression.^37^ A large meta-analysis of prospective cohort studies concluded that each key feature of insomnia, except for early-morning awakening, is associated with an increased risk of future CVD including stroke.^38^ In a recent analysis of the INTERSTROKE cohort, difficulty getting sleep or maintaining sleep was associated with a 30% increased stroke risk.^39^ An ancillary study to the CARDIA Study revealed insomnia being associated with a 23% higher fasting glucose level and a 48% higher fasting insulin level.^40^ In line, Spiegel et al.^41^ found that 1 week of reduced sleep (i.e. <4 h) aggravated the long-term risk profile of otherwise healthy young adults. Furthermore, a recent study of 1413 participants without hypertension or sleep apnea at baseline showed an association between insomnia with objective (polysomnography confirmed <6 h of sleep), but not subjective short-sleep duration and incident hypertension after a median follow-up of 5.1 years.^42^

Hypersomnia (sleep duration >9 h not attributable to other sleep disorders) is independently associated with stroke of microangiopathic origin.^43–45^ The U-shaped relationship between sleep duration and stroke risk was recently strengthened by Wang et al.^46^ reporting that both short- (⩽4 h) and long-sleep duration (⩾9 h) are associated with stroke risk and mortality. Patients with long sleep have a substantially elevated risk of diabetes, atrial fibrillation, inflammation, blood pressure variability, and increased arterial stiffness. The association between long sleep and stroke is independent of confounders, has a dose–response relationship, and is specific for stroke.^47^

Circadian rhythm sleep–wake disorders (CRSWD), caused by alteration of the endogenous circadian timing system or misalignment between ones intrinsic rhythm and the required sleep–wake cycle, are known to be associated with CVD.^8^ The Nurses’ Health Study identified an increased risk of stroke in shift-working nurses.^48^ In line, a meta-analysis by Vyas et al.^49^ enveloping more than 2 million people reported shift work being associated with stroke risk (OR = 1.05 (1.01–1.09) even after accounting for confounders. Furthermore, there is a known circadian variation in stroke onset with meta-analyses reporting an excess risk of stroke between morning hours and noon (+50%) and a decrease during night sleep hours, suggesting a protective role of sleep.^50–53^

Concerning the involved pathomechanisms responsible for the relationship between insomnia/sleep duration/CRSWD and stroke, elevated cortisol levels (reflecting a negative-feedback control of the hypothalamo–pituitary–adrenal axis) result in insulin resistance.^54,55^ Furthermore, as a systemic review by Irwin et al.^56^ revealed elevated CRP and interleukin (IL)-6 levels in such individuals, inflammation, a well-known risk factor for CVD, may play a role. CRSWD and short-sleep duration have also been associated with increased visceral fat potentially resulting in obesity and diabetes mellitus.^57,58^

## Sleep disorders after stroke

A meta-analysis of sleep quality after stroke indicates that poor sleep quality affects 53% of stroke patients.^59^ In 2020, a European task force of sleep and stroke researchers emphasized the necessity for clear guidelines to identify, adequately diagnose, and manage sleep disorders in stroke patients, prompting important investigations.^3^ The Sleep-disordered breathing in transient ischemic attack (TIA)/Ischemic stroke and continuous positive airway pressure (CPAP) treatment efficacy study conducted by Miano et al.^60^ reports severe changes of sleep architecture in individuals with stroke or transient ischemic attack, with sleep efficiency and REM sleep being affected the most. They also demonstrated a loss of cardiac autonomic dynamics during sleep after stroke in addition to diurnal variation of clock gene expression and sleep–wake rhythm biomarkers.^61,62^ Corresponding changes are also seen in animal models of stroke; in middle-aged C57BL/6 J mice with induced M1 occlusion, compared with sham mice, sleep latency and daytime sleepiness were increased with reduced non-REM sleep duration and disruption in sleep architecture.^63^

### SRBD

After stroke, the prevalence of SRBD increased to approximately 50–60% with about 30% of patients having severe OSA (apnea–hypopnea index >30/h). The prevalence of OSA did not decrease within the first 2 years after stroke.^64^ Even though the occurrence of OSA is unrelated to a specific infarct lesion, poststroke dysphagia and altered sleep position throughout the night (increased supine sleeping in stroke patients) are associated with OSA. On the contrary, poststroke central sleep apnea can be temporary and may manifest after brainstem stroke.

### RLS and PLMS

Unilateral, of the paralyzed limb, or bilateral poststroke RLS has been reported in 2.3–15.1% of stroke survivors.^65–69^ It has been associated with infarcts in the body of caudate nucleus, the lenticulo capsule, corona radiate, and ventral brainstem.^69,70^ The prevalence of RLS did not change in the acute and chronic phase of stroke patients.^71^ Compared with RLS, the evidence on poststroke PLMS is scarce.^3^ One prospective polysomnographic study could not find a difference in PLMS frequency in stroke patients compared with controls while a meta-analysis found that periodic limb movement (PLM) index after stroke is increased compared with controls.^72,73^

### Central disorders of hypersomnolence (Narcolepsy)

Even though case reports exist proclaiming that specific ischemia affected areas are associated with clinical symptoms of narcolepsy, the prevalence of poststroke narcolepsy seems to be identical to that of the general population.^74–77^

### RBD

Concerning poststroke RBD, only case reports exist.^78,79^ Anatomically, both cases described by Reynolds and Roy^78^ and Xi and Luning^79^ suffered pontine infarction. Whether such associations are causal remains uncertain. One hypothesis states that if brainstem nuclei involved in the control of muscle tone during REM sleep are affected, poststroke RBD may present. However, Tang et al.^80^ performed a polysomnographic study of patients with brainstem infarction revealing a reduced time spent in REM with preserved atonia, rendering the anatomic relationship inconclusive.

### Insomnia, sleep duration, and CRSWD

Insomnia has been reported in up to 40% of stroke survivors, which is markedly more frequent than in the general population.^81^ A recent prospective study delivered robust data on the frequency and evolution of sleep–wake disturbances in poststroke individuals.^82^ The authors report excessive daytime sleepiness, fatigue, or insomnia being evident in 14%, 28%, or 28% of cases, respectively, over a follow-up period of 3 years. Over time, insomnia and excessive daytime sleepiness showed improvement while fatigue remained stable.^71^ Poststroke insomnia is associated with other symptoms associated with worse outcome in stroke survivors, namely depression.^83^ Concerning treatment of insomnia in stroke patients, only few small-scale studies are available. A parallel group randomized controlled trial by Fleming et al.^84^ found a favorable effect of digital cognitive behavioral therapy on sleep after stroke but did not report data on its effect on stroke recurrence. Accordingly, antidepressants like mianserin have the potential to improve poststroke sleep but whether this affects stroke risk is unknown.^85^ Compared with benzodiazepines, nonbenzodiazepine hypnotics in treatment of insomnia were associated with reduced stroke risk over time in retrospective observational data.^86^ Huang et al.^87^ described a potential dose–response relationship of benzodiazepine use to stroke risk, favoring lower doses and less frequent intake. Overall, benzodiazepines are not recommended for treatment of sleep disorders in stroke patients as at high dose they have been associated with an increased risk of stroke recurrence, worse cognition, and SRBD.^3^

## Effect of sleep disorders on poststroke rehabilitation and outcome

Two small studies report less favorable outcome of stroke if RLS is present.^88,89^ Insomnia and related symptoms were associated with burden on daily living, health-related quality of life, and worse functional recovery in stroke survivors.^80,90,91^ A recent Mendelian randomization study showed a genetic liability to insomnia with sleep duration further being associated with an increased poststroke functional dependency (modified Rankin Scale (mRS) ⩾ 3).^92^ OSA is also related to a high risk of mortality and poor functional outcome after stroke. According to data derived from mouse models, sleep is a major, modifiable factor in regaining cognitive and motor function poststroke. de vivo et al.^93^ provided insights on the role of sleep on synaptic plasticity, as sleep, according to the authors, played a role in synaptic strengthening. Zunzunegui et al.^94^ reported that extracellular waste removal, an essential mechanism in stroke recovery, is impaired in mice with disrupted sleep. These valuable preclinical data, however, have yet to prompt large-scale clinical studies. Moreover, the impact of increased sleep duration (i.e. hypersomnia), CRSWD/sleep–wake cycle disruption on poststroke outcome have not been investigated yet. The same applies to the effect of treatment of sleep disorders on stroke recovery or stroke recurrence.^82^

## Take-home messages for clinicians and future perspectives

**Figure 1.**
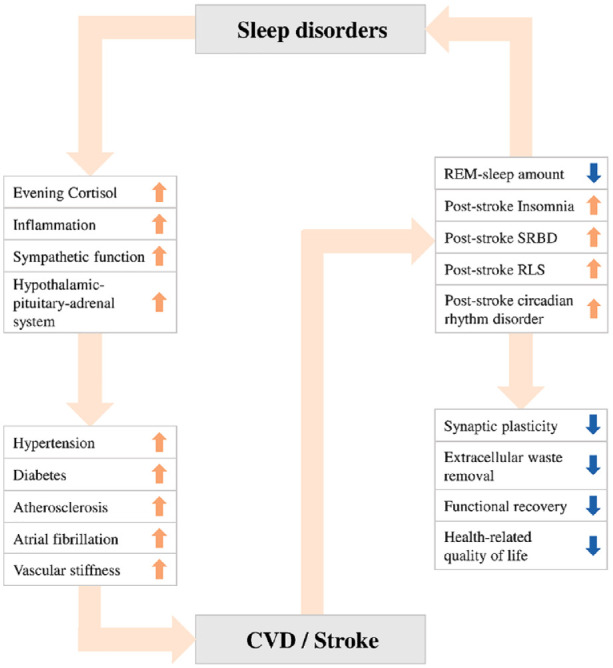
The link between sleep disorders and stroke.

The link between sleep disorders and stroke is compelling (Figure 1).

OSA is a relevant risk factor for stroke with its treatment potentially reducing its detrimental effect on poststroke outcome and stroke risk. Still, European but not American guidelines recommend screening and treatment of OSA in the acute phase of stroke.^3,4^ In a recent review, Baillieul et al.^4^ suggest the establishment of systematic screening with easy-to-use techniques in poststroke care with one ongoing study testing whether that should be done in the first days at the stroke unit (NCT03812653). Extending on such investigations and the data presented within this review, the authors support screening for nonrespiratory sleep disorders in stroke patients as well. This may enable the elucidation of pathomechanistic pathways which have to date been hampered through the following factors. First, the relationship between sleep and stroke is complex, particularly because sleep disorders are often co-morbid with other internal, neurological, and psychiatric diseases as well as the potential to co-existing OSA.^8,95^ Currently, available data mostly stems from observational questionnaire-based studies ensuing a high risk of bias. Second, large-scale studies with adequate screening tools for individual sleep disorders remain scarce. Third, treatment methods of nonrespiratory sleep disorders poststroke and whether current sleep guideline-recommended treatments, including the use of benzodiazepines in insomnia, relate to poststroke outcome are understudied.^3,96^ A shared statement of the European academy of neurology (EAN)/ the European respiratory society (ERS)/ the European sleep research society (ESRS) and the European stroke organization (ESO) concluded that potential future studies should feature the effect of treating poststroke insomnia on poststroke outcome and risk while taking comorbidities (e.g. depression) and treatment type (i.e. psychotropic drugs) into account. An overview of currently ongoing observational and interventional studies is given in Table 1.

**Table 1.** Selection of currently recruiting studies investigating the relationship of sleep and stroke (reference clinicaltrials.gov).

ClinicalTrials.gov identifier	Investigation	N, year completed
Observational		
NCT03274505	Prevalence of sleep disorders in transient ischemic attack and stroke.	N = 200, 2023
NCT05242393	Association of circadian rhythm disorder and outcome 14 days poststroke.	N = 250, 2024
NCT05012605	Impact of non-OSA sleep disorders on ADL, functional mobility, and recovery of stroke patients.	N = 200, 2025
NCT04312126	Investigate the role of memory replay during wakeful rest and sleep (naps) in retaining newly learned skills poststroke.	N = 138, 2025
NCT05746260	Association of sleep disruption and clinical motor outcomes.	N = 150, 2027
Interventional		
NCT03812653	Whether CPAP treatment for sleep apnea initiated acutely after stroke improves functional outcome and reduces stroke recurrence.	N = 3062, 2023
NCT05867290	Effect of mindful music listening on subjective and objective insomnia symptoms as well as mood and fatigue poststroke.	N = 6, 2023
NCT04876001	Effect of nurse-led brief behavior therapy for poststroke insomnia.	N = 60, 2023
NCT05511285	Efficacy of digital cognitive behavioral therapy in comparison with usual care alone for reducing insomnia symptoms after stroke.	N = 100, 2024
NCT05247125	Influence of combined blue light exposure and melatonin therapy on molecular biomarkers of circadian rhythms, sleep characteristics, and stroke outcome in acute stroke patients.	N = 80, 2024
NCT05623137	Effect of transcutaneous electrical nerve stimulation at acupoints on sleep quality, motor function, and cognition in older adult participants with chronic stroke.	N = 70, 2024
NCT02554487	Effect of early treatment of sleep apnea with adaptive servoventilation on the evolution of stroke lesion volume and clinical outcomes.	N = 201, Unknown
NCT05289518	Effect of remote ischemic conditioning on stroke-related insomnia.	N = 136, Unknown
NCT05170386	Effect of cognitive training on poststroke sleeping disorders.	N = 40, Unknown

OSA: obstructive sleep apnea; CPAP: continuous positive airway pressure; ADL: activity of daily living.

Large-scale studies based on systematic screening of sleep disorders in stroke patients using stringent inclusion criteria are needed and should go beyond SRBD. To enable such endeavors, the application of new technologies (i.e. wearables/nearables, automated sleep analysis, or artificial intelligence-based analysis methods) and novel biomarkers such as hypoxic stress,^97^ odds-ratio-product quantifying sleep quality,^98^ or hypnodensity (a neural network generated hypnogram compounding the entire information collected during sleep delivering more detailed analyses of sleep trends compared with sleep stage scoring) may be of particular value.^99^

In conclusion, sleep disorders may have a role in stroke prevention and poststroke management. Still, due to the currently limited available data, clear recommendations on screening methods and the effect of treatment of sleep disorders on poststroke outcome and incident stroke risk should be a focus of future research.

## Search strategy and selection criteria

Search terms “sleep,” “insomnia,” “narcolepsy,” “restless legs syndrome,” “periodic limb movements during sleep,” “excessive daytime sleepiness” AND “stroke” OR “cerebrovascular” in PubMed. English articles from 1990 to March 2023. Publications recommended by senior authors (MK, BH, AH, and SK) and cited by key articles were added. Furthermore, search terms “stroke” and “sleep” applied to ClinicalTrials.gov database.
